# Dance emotion recognition based on linear predictive Meir frequency cepstrum coefficient and bidirectional long short-term memory from robot environment

**DOI:** 10.3389/fnbot.2022.1067729

**Published:** 2022-11-11

**Authors:** Dianhuai Shen, Xiaoxi Qiu, Xueying Jiang, Dan Wang

**Affiliations:** ^1^College of Music and Dance, Huaqiao University, Xiamen, China; ^2^College of Education, Xiamen Nanyang Vocational College, Xiamen, China; ^3^School of Public Policy and Management, Tsinghua University, Beijing, China; ^4^Department of Computer Science, Heilongjiang University of Science and Technology, Harbin, China

**Keywords:** dance emotion recognition, robot environment, linear prediction coefficient, Meier frequency cepstrum coefficient, bidirectional long short-term memory, SVM

## Abstract

Dance emotion recognition is an important research direction of automatic speech recognition, especially in the robot environment. It is an important research content of dance emotion recognition to extract the features that best represent speech emotion and to construct an acoustic model with strong robustness and generalization. The dance emotion data set is small in size and high in dimension. The traditional recurrent neural network (RNN) has the problem of long-range dependence disappearance, and due to the focus on local information of convolutional neural network (CNN), the mining of potential relationships between frames in the input sequence is insufficient. To solve the above problems, this paper proposes a novel linear predictive Meir frequency cepstrum coefficient and bidirectional long short-term memory (LSTM) for dance emotion recognition. In this paper, the linear prediction coefficient (LPC) and Meier frequency cepstrum coefficient (MFCC) are combined to obtain a new feature, namely the linear prediction Meier frequency cepstrum coefficient (LPMFCC). Then, the combined feature obtained by combining LPMFCC with energy feature is used as the extracted dance feature. The extracted features are input into the bidirectional LSTM network for training. Finally, support vector machine (SVM) is used to classify the obtained features through the full connection layer. Finally, we conduct experiments on public data sets and obtain the better effectiveness compared with the state-of-art dance motion recognition methods.

## Introduction

Dance emotion recognition is widely used in artificial intelligence. For example, in aerospace, detecting negative emotional changes in astronauts can provide timely psychological counseling. In terms of humanized telephone service, automatic customer service system and manual service can be selected according to customer’s mood change (Yin et al., 2021; Chen, 2022). In the aspect of quality teaching, teachers adjust the course and difficulty of class appropriately by observing students’ learning emotions. In terms of medical treatment, by observing the mood of patients with mania or depression, doctors can be informed in time to help eliminate bad emotions.

**FIGURE 1:**
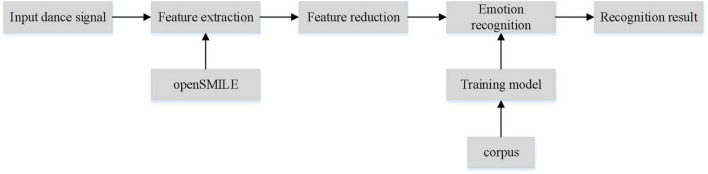
Dance emotion recognition system block diagram.

Dance emotion recognition includes feature extraction, feature dimension reduction, emotion classification and other main parts (Luo et al., 2021). The system block diagram of the recognition process is shown in Figure 1. (1) Feature extraction: it refers to the preprocessing of the collected motion signal by analog and digital processing technology and the application of hardware or software technology. Then it extracts the acoustic features that can represent the emotion through feature extraction tools. (2) Feature dimension reduction: it refers to the dimensionality transformation of extracted features to remove redundant information and extract significant features that can represent dance emotion. (3) Emotion classification: it refers to the process of establishing the emotion recognition model of dance, and matching the test set with the recognition model to get the emotion type of dance. Feature extraction and emotion classification are the key technologies in dance emotion recognition. The quality of dance feature extraction directly affects the recognition results, and a good corpus can also improve the recognition performance (Guimond et al., 2022).

### Features of dance emotion

Dance feature is an important factor in motion emotion recognition, and feature extraction is a key step in dance emotion recognition. Dance features can be divided into continuous speech features, sound quality features, spectral-based features and non-linear teager energy operator (TEO)-based features. Common spectral features include Linear Prediction Cepstral Coefficient (LPCC) (Krobba et al., 2020), Mel Frequency Cepstral Coefficient (MFCC) (Albadr et al., 2021), Log Frequency Power Coefficient (LFPC) (Gao, 2022). LPCC linearly approximates speech at all frequencies, which is inconsistent with human auditory characteristics. The MFCC focuses on the auditory properties of the human ear because the level of sound heard is not linearly proportional to frequency. The MFCC first maps the linear spectrum to the Mel non-linear spectrum based on the auditory properties and then converts it to cepstrum. Relationship between Mel frequency and actual frequency is:


(1) $$Mel\,\left(f\right)=2595\,\mathrm{lg}\left(1+\frac{f}{700}\right)$$

where *f* is the actual frequency of the speech signal.

Wang et al. (2022), the multi-resolution idea of wavelet analysis is combined with different forms of TEO and MFCC, and five non-linear features are proposed for speech emotion recognition. Qadri et al. (2022) proposed that Teager-energy based MFCC (TEMFCCs) was classified on Berlin database by Gaussian mixture model (GMM), and experimental results showed that TEMFCCs had better performance than MFCC. In order to facilitate the subsequent speech emotion recognition, the speech processing tool OpenSMILE is used to extract the speech features, and the extracted features are saved as .csv files.

### Emotion feature dimension reduction

The classical dimension reduction methods can be divided into linear dimension reduction methods and non-linear dimension reduction methods. Linear dimensionality reduction methods include principal component analysis (PCA) (Roland et al., 2021), linear discriminant analysis (LDA) (Zhu et al., 2022), locality preserving projections (LPP). Non-linear dimensionality reduction methods can be divided into manifold learning, neural network-based method and kernel based method. The kernel based method has kernel PCA. There are three methods based on manifold learning: isometric feature mapping (ISOMAP) (Moradzadeh et al., 2020), multidimensional dimension transformation (MDT) (Leprince et al., 2021), local linear embedding (LLE), laplacian eigenmaps (LE), etc. The neural network methods include autoencoder networks (AN) (Jin et al., 2021) and self-organizing feature mapping (SOM) (Ghahramani et al., 2021). Different principles and structures of each dimension reduction algorithm will bring different recognition effects. The comparison is shown in Table 1.

**TABLE 1:** Advantages and disadvantages of various dimensionality reduction methods.

Method	Advantage	Disadvantage
PCA	Simple concept, convenient calculation and optimal linear reconstruction error	After dimensionality reduction, there is no definite rule for dimensionality selection, which can not deal with non-linear data and complicated calculation
LDA	Supervise dimension reduction, which can be used to categorize tasks	It is not suitable for dimensionality reduction of non-Gaussian distribution samples, which may over-fit the data and reduce the dimensionality to the k^–1^ dimension at most
LLE	Less variable parameters, translation, rotation and other invariance, retain the inherent structure of data, short loop is not sensitive to ISOMAP	The data samples are required to be dense and locally linear, and the excessive selection of parameters k and d will affect the effect of dimension reduction and be sensitive to noise
ISOMAP	It can extract the features with strong identification ability, and use the manifold measuring line distance instead of Euclidean distance to better retain the geometric structure of data	With topological instability, the influence of short loop requires the manifold to be convex, otherwise deformation will occur, and the problem of holes in the manifold cannot be solved
LE	Local feature retention makes it less sensitive to isolated points and noise	The classification information between samples will be ignored when calculating the Euclidean distance of samples
T-SNE	Define soft boundaries of local and global structures	Large amount of calculation and long calculation time
MDS	Better retention of differences between data	High dimensional non-linear data cannot be processed, and there is no unified standard to evaluate the quality of embedded dimension
KPCA	PCA reconstruction in nuclear space is simple and can deal with non-linear data outside PCA	The practical significance of extraction index is not clear, the calculation amount is larger than PCA, and the projection of test sample on space vector is complex
LPP	The practical significance of extraction index is not clear, the calculation amount is larger than PCA, and the projection of test sample on space vector is complex	Susceptible to small sample size problems and failing to consider available supervisory information
LTSA	It is a good reflection of local geometry	Unable to process large sample data, unable to effectively process new data
NN	Automatic learning of good features in data	Many iterations are required, and the calculation is complex, so the application will be limited

LTSA, local tangent space alignment; MDS, mulitiple dimensional scaling; KPCA, kernel PCA; T-SNE, *t*-distributed stochastic neighbor embedding.

### Related emotion recognition algorithms

At present, most dance emotion recognition algorithms can be divided into two categories: single-based classifier and hybrid-based classifier. Single-based classifier can be divided into linear classifier and non-linear classifier. Linear classifiers include naive Bayes classifier (NBC), neural networks (NN), etc. Non-linear classifiers include hidden Markov model (HMM), GMM, K-nearest neighbor (KNN), decision tree (DT) and Softmax classifier. The most typical combination-based classifiers are Boosting, Bagging, and Random Forest (RF). With the development of research, the recognition effect of using single classifier has certain limitations.

At present, many researchers have devoted themselves to the research of multi-classifier system for dance emotion recognition. Bhosle and Deshmukh (2019) and Pan and Yang (2021), the fusion of KNN, radial basis function (RBF), and Bayesian network was proposed, and the accuracy reached 71.40%. Jacob and Mythili (2015) proposed to connect NBC in layers to extract prosodic features such as pitch, energy, duration, and zero crossing rate. In the case of two-layer classifier connection, the recognition rate reached 83.5%, and in the case of three-layer classifier connection, the recognition rate reached 88.8%.

In addition, Li et al. (2022) proposed the concept of ensemble learning, that is, training multiple classifiers according to data samples to complete classification tasks. These classifiers have certain complementary functions and can improve the generalization ability of the system while reducing errors. Albadr et al. (2021) extracted MFCC, line spectral frequencies (LSF), polymeric ferroelectric liquid (PFL) and other features from linguistic data consortium (LDC) emotion database, and integrated KNN classifier with Bagging algorithm, thus improving the recognition rate. Lai et al. (2020) extracted significant prosodic features from SAVEE database and used RF algorithm to identify emotional labels, with an average recognition rate of 66.28%. Under the same experimental conditions, the average recognition rate was improved by 13.78% compared with the linear discriminant analysis algorithm. Compared with deep neural network, the average recognition rate was improved by 6.58%. However, due to the high computational complexity of the above methods, the extraction efficiency of these methods does not improve much when complex features are encountered.

To solve the above problems, we propose a novel dance emotion recognition based on linear predictive Meir frequency cepstrum coefficient and bidirectional long short-term memory (LSTM). The new model can fully extract the dance emotion feature. Meanwhile, it also reduces the amount of calculation. Experimental results show that the proposed model achieves 93.45% recognition performance in public data sets.

This paper is organized as follows. In section “Related works,” we give the related works for this paper. Section “Proposed dance motion recognition method” detailed introduces the proposed dance emotion recognition model. Experiments and analysis are conducted in section “Experiments and analysis.” There is a conclusion in section “Conclusion.”

## Related works

In recent years, emotion recognition, as an important medium of human-computer interaction, has attracted more and more attention from researchers. Human emotion has always played an important role in human communication. Emotion recognition refers to the analysis of the emotional changes hidden in human conversation, which can identify the possible emotional changes of speakers by extracting the relevant features of speech and putting them into neural network for classification (Jiang and Yin, 2021). In reality, emotion recognition has a wide range of application scenarios, such as customer service personnel in the process of telephone communication with customers, through the emotion recognition system to track the customer’s mood changes in real time, so as to provide quality service more actively. Since the expression of emotion depends on many factors, such as the speaker’s gender, age, dialect, etc., a major challenge for researchers is how to better extract distinguishing, robust, and significantly influential features to improve the model’s recognition ability.

At present, feature extraction methods are mainly divided into two categories: one is to manually extract short-term features from audio signals, such as Meir cepstrum coefficient, pitch and energy, and then apply short-term features to traditional classifiers, such as GMM, matrix decomposition and HMM, etc. The other is automatic feature extraction using NN, such as Convolutional Neural Network (CNN) (Feng, 2022), auto-encoder, Recurrent Neural Network (RNN) (Ackerson et al., 2021), LSTM (Liu et al., 2022), CNN + LSTM, etc. Gao et al. (2021) and Mandić (2022) show that these methods have achieved good results in speech classification tasks.

With the improvement of artificial intelligence and hardware computing power, deep learning methods are widely used in audio classification. Deep learning has excellent learning and generalization abilities, and can extract task-related hierarchical feature representation from a large number of training samples. It has achieved great success in the research work of automatic speech recognition and music information retrieval. Huang et al. (2014), CNN was first used to learn the salient features of dance emotion, and its excellent performance was demonstrated in several benchmark data sets. Jiang et al. (2020), one-dimensional CNN was used to preprocess the audio samples in order to reduce noise and emphasize specific areas of the audio file. Since audio signals could transmit contextual information in the time domain, that is, the audio information at the current moment was related to the information at the previous moment, RNN and LSTM could be applied to capture the time-dependent feature representation in emotion recognition task. Alhagry et al. (2017) proposed a emotion recognition method combining frame-level speech features with attention and LSTM, which could extract frame-level speech features from waveform to replace traditional statistical features, so as to maintain the internal temporal relationship of original speech through frame sequence. Cui et al. (2020), CNN and LSTM were combined to mine the spatio-temporal features of input sequences, which was also a common processing method in speech emotion classification tasks. Jiang et al. (2019), a convolutional RNN based on attention mechanism was further proposed, and mel-spectrogram was used as the input, which effectively improved the recognition ability of the model. Li et al. (2020) used Bidirectional LSTM model and attention-based CNN to build a network for learning features. It combined with VGG16 for mel-spectrum pretreatment, achieving a high recognition accuracy. But the model size was relatively large, increasing the difficulty of training. These models demonstrate the effectiveness of the combination of attention mechanism and neural network. According to the characteristics of dance emotion data set, linear prediction Meier frequency cepstrum coefficient (LPMFCC) is used as feature extractor. The extracted features are input into the bidirectional LSTM network for training. Support vector machine (SVM) is a task classifier, and the fusion of the above approaches can improve the classification effect. This paper also conduct a comparative experiment between SVM and other two classifiers. The experimental results show that the end-to-end model based on LPMFCC and SVM is very suitable for dealing with dance emotion recognition problem and can improve the model recognition performance.

## Proposed dance motion recognition method

In this section, we detailed introduce the new dance motion recognition method including LPMFCC and Bi-LSTM.

### Linear prediction Meier frequency cepstrum coefficient for feature extraction

Linear prediction is a common method for motion analysis. It can not only get the prediction waveform of dance signal, but also provide a very good channel model. The main idea is that in view of the correlation between the sampling points of the dance signal, the sampled values of the speech signal at a certain time can be approximated by the linear combination of the sampled values at the previous time, so as to estimate and predict the waveform of the dance signal. In order to determine the LPC of dance samples, it is necessary to minimize the mean square error between the linear prediction sample value and the actual dance sample value. The LPC reflects the characteristics of dance signal.

According to the above analysis, the LPC is calculated. After preprocessing the dance signal, the p-order linear prediction is to predict the sampling value *s*(*n*) at this moment by using the linear combination of the sampling value {*s*(*n*−1),*s*(*n*−2),⋯,*s*(*n*−*p*)} at the previous *p* times of the dance signal. The obtained prediction signal $\hat{s}\,\left(n\right)$ is:


(2) $$\hat{s}\,\left(n\right)=\sum_{k=1}^{p}a_{k}\,s\,\left(n-k\right)$$

where *a*_*k*_ is the LPC, and its linear prediction error is:


(3) $$e\,\left(n\right)=s\,\left(n\right)-\hat{s}\,\left(n\right)=s\,\left(n\right)-\sum_{k=1}^{p}a_{k}\,s\,\left(n-k\right)$$

In order to optimize the prediction effect, it is necessary to minimize the mean square value of the prediction error. The mean square value of the prediction error is:


(4) $$\varepsilon =E\,\left[e^{2}\,\left(n\right)\right]$$

In order to minimize the mean square value of the prediction error, it is necessary to take the partial derivative of the mean square value of the prediction error and make it zero, as shown in Eq. 5.


(5) $$\frac{\partial \left[e^{2}\,\left(n\right)\right]}{\partial a_{k}}=0,k=1,2,\cdots ,p$$

We can obtain:


(6) $$s\,\left(n-i\right)\,\left(n\right)=\sum_{k=1}^{p}a_{k}\,s\,\left(n-k\right)\,s\,\left(n-i\right)$$

If we define:


(7) ϕ⁢(i,k)=s⁢(n-i)⁢s

Then Eq. 6 can be changed into Eq. 8.


(8) $$\phi \,\left(i,0\right)=\sum_{k=1}^{p}a_{k}\,\phi \,\left(i,k\right)$$

Obviously, the LPC *a*_*k*_ can be obtained by solving the equations obtained by Eq. 8. In this paper, the auto-correlation method and Levinson-Durbin recursive method are used to solve these equations.

Linear prediction Meier frequency cepstrum coefficient is a new characteristic parameter combining LPC and MFCC. LPC parameters reflect the linear characteristics of speech, but have the disadvantage of being greatly disturbed by environmental noise. The MFCC parameters reflect the non-linear characteristics of dance, and transform the actual frequency of dance to the Merle frequency that conforms to the auditory characteristics of human ear. When the actual frequency is less than 1 kHz, the relationship between Merle frequency and actual frequency is approximately linear. When the actual frequency is greater than 1 kHz, the relationship between the Merle frequency and the actual frequency can be approximated as a pairwise number. The general expression of the relationship between Merle frequency and actual frequency is:


(9) $$f_{mel}=2595\cdot {\text{log}}_{10}*(1+f/700)$$

where *f*_*mel*_ stands for Mel frequency and *f* stands for actual frequency.

MFCC parameters are relatively sensitive to the low frequency part of dance. However, ambient noise is in the high frequency part of dance Therefore, MFCC parameters have strong anti-interference ability and good robustness to environmental noise. The LPMFCC parameter is actually the LPC cepstrum parameter that converts the LPC parameter into Meyer frequency.

The LPMFCC feature extraction first needs to extract the LPC coefficient of dance. After preprocessing the dance signal *x*(*n*) with pre-weighting, framing, and windowing, the LPC coefficient *x*_*a*_(*n*) of each dance is calculated. The order of the LPC coefficient must be equal to the number of voice samples in a frame. Secondly, cepstrum calculation of LPC coefficients on Mayer frequency is carried out. Fourier transform of LPC coefficients is first carried out, then the corresponding discrete spectrum *X*_*a*_(*k*) of LPC coefficients is obtained through discrete fourier transformation (DFT), that is,


(10) $$X_{a}\,\left(k\right)=\sum_{n=0}^{N-1}x_{a}\,\left(n\right)\,e^{-j\,2\,p\,n\,k/N},0\leq k\leq N-1$$

We take to square amplitude spectrum calculation for it and get the discrete energy spectrum |*X*_*a*_(*k*)|^2^. Where N is the point number of the Fourier transform. Then, a set of Meir scale triangular filters are used to filter the discrete energy spectrum, and the logarithmic operation is performed on the output results to obtain the logarithmic energy *Z*_*a*_(*m*), the equation is as follows.


(11) $$Z_{a}\,\left(m\right)=I\,n\,\left(\sum_{k=0}^{N-1}{\left|X_{a}\,\left(k\right)\right|}^{2}\,H_{m}\,\left(k\right)\right),0\leq m\leq M$$

The *H*_*m*_(*k*) is a number of band-pass filters, and M is the number of filters. Finally, the logarithmic energy is calculated by discrete cosine transform, and the new characteristic parameter LPMFCC is obtained.


(12) $$C_{a}\,\left(n\right)=\sum_{m=0}^{M-1}Z_{a}\,\left(m\right)\,\cos\left[\frac{p\times n\,\left(m+0.5\right)}{M}\right]$$

**FIGURE 2:**
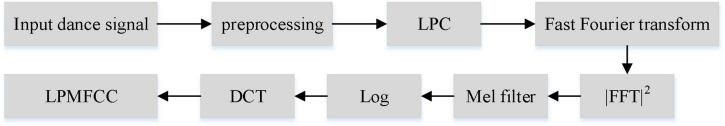
Feature extraction process of LPMFCC.

In summary, it can be seen that the calculation method of LPMFCC refers to the calculation of MFCC. It carries out cepstral operation on LPC coefficient at Meir frequency. The specific extraction process is shown in Figure 2. In addition, the LPMFCC feature *Y*_*i*_(*i* = 1,⋯,*T*) extracted from dance signal *S*_*i*_ is denoted as *Y* = {*Y*_1_,⋯,*T*_*T*_}. The average feature vector $\hat{Y}$ is used to represent the features of dance signal S, where $\hat{Y}=\frac{1}{T}\,\sum_{t=1}^{T}Y_{i}$ and T represent frame number of dance signal S.

### Bi-long short-term memory for feature training

With the continuous development of deep learning, the structure of NN is becoming more and more complex. Compared with the simple feed-forward NN in the past, there are both feed-forward and internal feedback connections between the hidden layers of RNN. RNN can process sequence data well, but there are problems of gradient disappearance and gradient explosion. The gated recurrent unit in LSTM can solve the gradient problem well, but LSTM only uses the previous moment information, so the prediction result is not very accurate. Bi-LSTM makes use of both past and future information (Marini et al., 2021), so that the prediction results of the network are more accurate. Therefore, Bi-LSTM is selected in this study to extract dance emotion time series features.

**FIGURE 3:**
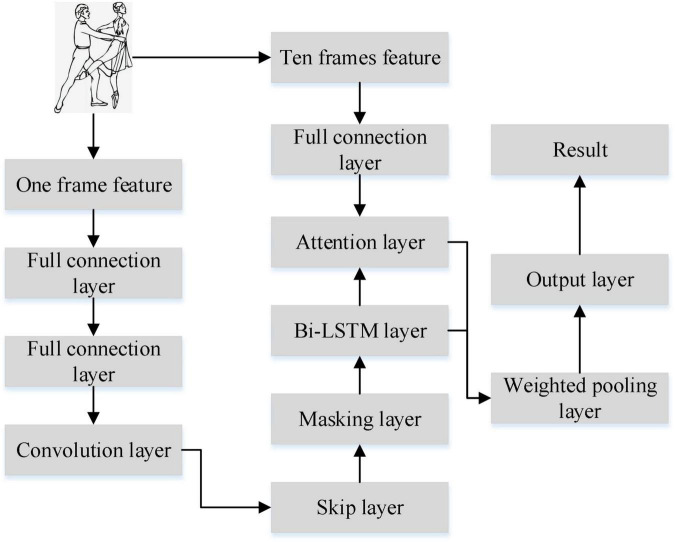
SA-Bi-LSTM network structure.

Only extracting time series information cannot represent dance emotion well. Therefore, convolution operation is used to extract dance spatial information in this study. Combining temporal and spatial information to represent emotion can make the prediction result more ideal. The attention mechanism gives different attention to frame features from different moments, and reduces the computational burden greatly. Therefore, this study constructs a new model SA-Bi-LSTM for dance emotion recognition based on key technologies such as attention mechanism, skip connection and masking operation, as shown in Figure 3.

The model has 8 layers including 2 fully connected layers, convolution layer, jump layer, masking layer, Bi-LSTM layer, attention layer, and pooling layer. The full connection layer mainly extracts common features of dance signals. Convolutional layer extracts emotional spatial features of dance. The jump layer fuses the features extracted from the full connection layer with the features extracted from the convolution layer, and it solves the gradient problem well. The main function of the masking layer is to make the value 0 in the data not participate in the calculation and reduce the calculation amount. Bi-LSTM extracts the time series information of dance emotion. The attention layer assigns weights according to the contribution degree of different time series features to emotion. The pooling layer calculates the weight of the entire dance emotion sequence.

The output $h_{j}^{1}$ of the first fully connected layer of SA-Bi-LSTM model is calculated as follows:


(13) $$h_{j}^{1}=f\,\left(\sum_{i=1}^{d}w^{1}\,x+b\right)$$

where *b* = [*b*_1_,*b*_2_,⋯,*b*_36_]^*T*^ is the bias. *x* is the 36-dimensional input eigenvector, that is, *x* = [*x*_1_,*x*_2_,⋯,*x*_36_]^*T*^. $w_{i\,j}^{1}$ is the component of the weight matrix *w*^1^, which represents the weight matrix of the i-th node of the input layer connected to the j-th node of the first full connection layer. The weight matrix *w*^1^ is defined as $w^{1}={\left[w_{i\,j}^{1}\right]}_{i\times j}^{T}$. *f*(⋅) is the LeakyReLU function.

In this model, the convolution layer acts as a local feature extractor. When the original dance data is transferred to the convolution layer, it will carry out convolution operation with the convolution kernel, and then generate the feature graph through the dot product operation between the convolution kernel and the input. In the two-dimensional convolution layer, an input signal *x*(*i*,*j*) is convolved with the convolution kernel *w*(*i*,*j*) with size (*i*,*j*) to obtain *z*(*i*,*j*). In this paper, the random initialization is used to set the convolution kernel.


(14) $$\begin{array}{l} z(i,j)=x(i,j)\times w(i,j)\\ \;\;\;\;=\sum_{s=-a}^{a}\sum_{t=-b}^{b}x(s,t)\cdot w(i-s,j-t) \end{array}$$

Similar to human selective vision, the attention mechanism can sift through a large amount of information for important information. The attention mechanism works by assigning different numerical weights to each component of the input sequence *x* = [*x*_1_,*x*_2_,⋯,*x*_*n*_]. It gives more weight to important components and less weight to unimportant components. The weight of the component is obtained through the training model, and the conditional probability of the i-th component of the given training parameter matrix *W* and input sequence *x* is calculated by the Softmax function, which is mathematically expressed as:


(15) $$a_{i}=\exp\left(f\,\left(x_{i}\,W\right)\right)/\sum_{j=1}^{n}\exp\left(f\,\left(x_{j}\,W\right)\right)$$

where *f*(⋅) represents the scoring function, which is jointly determined by the training parameter matrix *W* and the input sequence *x*, namely,


(16) $$f\,\left(x,W\right)=W^{T}\,x$$

Then, the weighted average sum of the input sequence is calculated to obtain the attention value of the entire sequence, which is mathematically expressed as:


(17) $$a\,t\,t\,e\,n\,t\,i\,o\,n\,\left(x,W\right)=\sum_{i=1}^{n}a_{i}\,x_{i}$$

The output of the skip layer is used as the input of the masking layer. Masking operation *y*_*m*_ is calculated by:


(18) $$y_{m}=M\,\text{as}\,k\,\left(F_{c},0\right)$$

In the Eq. 18, the value 0 in *F*_*c*_ is excluded from calculation, which can reduce the amount of calculation.

The output of the masking layer is used as input to the Bi-LSTM layer. In Bi-LSTM layer, the input of the current moment *t* is $o_{c\,o\,n\,v}^{3}$, and the output *h*_*t*_ is:


(19) $$h_{t}=o_{t}\,\Theta \,\tanh\left(C_{t}\right)$$

(20) $$C_{t}=C_{t-1}\,\Theta \,f_{t}+{\tilde{C}}_{t}\,\Theta \,i_{t}$$

where *C*_*t*_ is the update state of memory unit at time *t*. *f*_*t*_ is the output of *t* time forgetting gate. *i*_*t*_ is the output of the input gate at time *t*. *o*_*t*_ is the output of the output gate at time *t*.

The input of the Bi-LSTM layer is used as the input of the attention layer. In the attention layer, the weight of attention parameters of each frame is calculated, namely:


(21) $$\alpha =S\,o\,f\,t\,\max\left(u\cdot y_{B}\right)$$

where, “.” stands for dot product operation. *u* is a 256-dimensional vector. *y*_*B*_ is the output of the Bi-LSTM layer and the probability is calculated by the Softmax() function. The eigenvalue corresponding to the maximum probability is the target that the attention mechanism should pay attention to.

The pooling layer receives input from the attention layer and calculates the weight *z*_*p*_ of each sequence, that is:


(22) $$z_{p}=\alpha \cdot y_{B}$$

At the output layer of SA-Bi-LSTM network model, probabilities are calculated and classified by Softmax() function:


(23) $$y_{n\,k}=S\,o\,f\,t\,\max\left(z_{p}\right)$$

To find the optimal weight and bias, the SA-Bi-LSTM network is trained using the cross entropy loss function. The cross entropy loss function *L*_*CE*_ can be expressed as:


(24) $$L_{C\,E}=-\frac{1}{N}\,\sum_{n}\sum_{k}t_{n\,k}\,\log y_{n\,k}$$

where N is the total number of samples. *n* is the n-th sample. *k* = 0,1,⋯,6 is the k-th class. *t*_*nk*_ is the sample label. *y*_*nk*_ is the output of SA-Bi-LSTM network and represents the probability that the n-th sample belongs to the k-th class.

To solve the problem of linear indivisibility of low-dimensional space, SVM uses kernel function to map data from low-dimensional sample space to high-dimensional feature space, and then seeks a hyperplane in the feature space to achieve linear indivisibility of samples. The non-linear separable SVM optimization problem can be described as:


(25) $$\min_{w,b,\xi }\left\{0.5\,{||w||}^{2}+C\,\sum_{i=1}^{L}\xi_{i}\right\}$$

(26) $$y_{i}\,\left\{w\cdot x_{i}+b\right\}\geq 1-\xi$$

where *x* is the feature vector. *y*_*i*_ ∈ [+ 1,−1] is the category label. *w* is the weight vector. *b* is the classification threshold vector. C is the penalty factor. ξ_*i*_ is the relaxation variable. L is the number of training samples. λ_*i*_ is Lagrange factor. *K*(*x*_*i*_,*x*) is the kernel function. Radial basis function is selected as the kernel function of the model in this paper, and the equation is as follows:


(27) $$K\,\left(x_{i},x\right)=\exp\left\{-\gamma \,{||x_{i}-x||}^{2}\right\}$$

### Weights updating

In this paper, the classical back-propagation error (BP) algorithm is used to update the weights of nodes at each layer of the proposed network. The main idea of back propagation is to use the error between the output result of neural network and the output layer to calculate and adjust the weight of the leading layer in the output layer. Then, the error estimate between the output of the neural network and the training target is used to update the connection weights of the NN in the previous layer. In this way, the error selection is modified layer by layer from the output to the input, and the connection weights of each neural network layer are obtained. The error of each layer is used to modify the corresponding connection weight matrix. The gradient descent algorithm is usually used for training. We define the output error of neuron *i* when it selects n generations as shown in Eq. 28


(28) $$e_{i}\,\left(n\right)=d_{i}\,\left(n\right)-y_{i}\,\left(n\right)$$

where *d*_*i*_(*n*) is the target output vector. *y*_*i*_(*n*) is the actual output vector of neuron *i*. If the error of neuron *i* is regarded as the instantaneous value of energy $0.5\,e_{i}^{2}\,\left(n\right)$, the sum of error energy of all neurons in the output layer is the total error value of the output neural network node. The calculation of the total error value *ξ*(*n*) is shown in Eq. 29:


(29) $$\xi \,\left(n\right)=0.5\,\sum_{i\in Y}e_{i}^{2}\,\left(t\right)$$

where Y is the number of neurons in the output layer.

Weight modification of the output layer: at time t, the modified value $\Delta \,W_{i\,j}^{3}\,\left(t\right)$ of the connection weight between the i-th neuron of the output layer and the j-th neuron of the hidden layer *z*(*t*) in the neural network is shown in Eq. 30:


(30) $$\Delta \,W_{i\,j}^{3}\,\left(t\right)=\eta \cdot \delta_{i}\cdot z_{j}\,\left(t\right)$$

where η is the learning rate and *z*_*j*_(*t*) is the input signal of j-th neuron in the output layer. δ_*i*_ is the local gradient, and the calculation method is shown in Eq. 31:


(31) $$\delta_{i}=e_{i}\,\left(t\right)\cdot f^{\prime }\,\left(y_{i}\,\left(t\right)\right)$$

*y*_*i*_(*t*) is the actual output of the i-th neuron in the output layer.

Modification of the hidden layer *z(t)*: at time *t*, the correction $\Delta \,W_{j\,q}^{2}\,\left(t\right)$ of the connection weights between the j-th neuron of the hidden layer *z(t)* and the q-th neuron of the hidden layer *x(t)* is shown in Eq. 32:


(32) $$\Delta \,W_{j\,q}^{3}\,\left(t\right)=\eta \cdot \delta_{j}\cdot x_{q}\,\left(t\right)$$

where, *x*_*q*_(*t*) is the input of the q-th neuron in the hidden layer. δ_*i*_ is the local gradient, and the calculation method is shown in Eq. 15:


(33) $$\delta_{j}\,\left(t\right)=f^{\prime }\,\left(z_{i}\,\left(t\right)\right)\cdot \sum_{i\in Z}\delta_{i}\cdot W_{i\,j}^{3}\,\left(t\right)$$

Modification of input layer *E(t)*: at time t, the correction $\Delta \,W_{j\,q}^{5}\,\left(t\right)$ of connection weights between the j-th neuron of hidden layer *z(t)* and the p-th neuron of input layer *E(t)* is shown in Eq. 34:


(34) $$\Delta \,W_{j\,q}^{5}\,\left(t\right)=\eta \cdot \delta_{j}\cdot e_{p}\,\left(t\right)$$

Modification of the weight of memory layer *x*_*c*_(*t*): at time t, the correction value $\Delta \,W_{q\,m}^{4}\,\left(t\right)$ of the connection weight between the q-th neuron of hidden layer *x(t)* and the m-th neuron of memory layer *x*_*c*_(*t*) is shown in Eq. 35:


(35) $$\Delta \,W_{q\,m}^{4}\,\left(t\right)=\eta \cdot \delta_{q}\cdot x_{c_{m}}\,\left(t\right)$$

where *x_*c_m_*_*(*t*) is the input of the m-th neuron in the memory layer of the network, δ_*q*_ is the local gradient, and the calculation method is shown in Eq. 36:


(36) $$\delta_{q}=f^{\prime }\,\left(x_{q}\,\left(t\right)\right)\cdot \sum_{i\in Q}\delta_{j}\,\left(t\right)\cdot W_{i\,q}^{2}\,\left(t\right)$$

## Experiments and analysis

Table 2 compares the performance of SA-Bi-LSTM network on the emotional corpus of dance under different dropout values.^1^ The Optimizer is Adam, Epochs = 200, Batch_size = 32. The number of model cross validation (K_folds) is 10. In the whole datasets, 60% for training, 20% for testing, and the remaining 20% for validation. The evaluation indexes include confusion matrix, accuracy, mean value, and variance.

**TABLE 2:** Performance comparison in ten model cross validation with SA-Bi-LSTM.

Dropout	1	2	3	4	5	6	7	8	9	10
0.7	66.47	59.92	64.60	63.66	60.86	64.60	62.73	59.92	48.71	58.99
0.6	63.66	63.66	64.60	69.27	66.47	66.47	66.47	72.07	59.92	66.47
0.5	72.07	66.47	67.40	59.92	70.20	67.40	64.60	69.28	57.12	68.33
0.4	71.14	69.27	64.60	72.07	69.27	73.01	70.20	73.01	53.38	72.07
0.3	73.01	68.33	68.33	73.01	65.53	69.27	69.27	68.40	61.79	72.07
0.2	71.14	67.40	67.40	70.20	73.01	73.01	67.40	74.88	60.86	68.33
0.1	72.07	67.40	71.14	74.88	73.01	67.40	69.27	72.07	59.99	76.75
0	72.07	68.33	67.40	74.88	65.53	70.20	66.47	75.81	59.92	69.27

The following conclusions can be drawn from Table 1.

First, the average performance of the model varies greatly for different dropout values. For example, when the dropout values are 0.1 and 0.7, the average recognition performance differs by nearly 10%, suggesting that the choice of dropout is critical to model performance.

Second, as the dropout value drops from 0.7 to 0.1, the average performance of the SA-Bi-LSTM model increases. This is because the larger the dropout value is, the more dance emotion information is lost, resulting in a lower recognition rate. But when the dropout value drops to 0, the model’s average recognition performance drops by 1.31%, because the model overfits the training data, making it difficult to predict the test data. The loss function of the model is small in the training data and the prediction accuracy is high, but the loss function is large in the test data and the prediction accuracy is low.

Finally, the performance of SA-Bi-LSTM model varies greatly in different model cross validation times. For example, when the dropout value is 0.1, the recognition performance can be as high as 76.75% and as low as 58.99% during 10 times of cross-validation. In the cross-validation of other models, the performance achieved is closer to 76.75%, so 58.99% is treated as an outlier in post-processing.

Table 3 shows the confusion matrix of the SA-Bi-LSTM model on a dance database. Each column represents the true category to which each type of sample belongs. Each row represents the probability that one type of emotion is predicted to be another. The numbers on the diagonal indicate the probability that the corresponding category is correctly identified. This confusion matrix has the following characteristics:

**TABLE 3:** Performance of SA-Bi-LSTM network model.

Dropout	Training set	Test set	ACC	Precision
0.7	66.47	66.47	66.47	71.03
0.6	73.24	72.07	73.01	75.65
0.5	89.13	72.07	85.72	85.89
0.4	92.17	73.01	88.33	87.72
0.3	94.97	73.01	90.58	92.31
0.2	94.97	74.88	90.95	91.68
0.1	96.37	76.75	92.45	94.11
0	89.13	75.81	88.90	89.01

First of all, the probability of correct recognition on the diagonal is more than 90.00%, indicating that each emotion category has achieved good recognition performance. For example, in the W emotion, 97.75% of the samples are correctly predicted, 0.90% of the samples are correctly predicted as A emotion, and 1.68% of the samples are correctly predicted as F emotion. That is, in 127 emotion samples, 124 samples are correctly predicted, and only 3 samples are predicted as other emotion, which is a very considerable recognition result. Secondly, although the recognition rate of F emotion reaches 90.25%, there is still a certain difference compared with W emotion. A total of 4.34% of the samples are predicted to be W emotion, indicating that F emotion and W emotion are easily confused. Finally, one type of sample may be easily predicted as another, but the reverse is not necessarily true. For example, 2.64% of the samples of N emotion can be easily predicted as A emotion, but A emotion cannot be predicted as N emotion.

**FIGURE 4:**
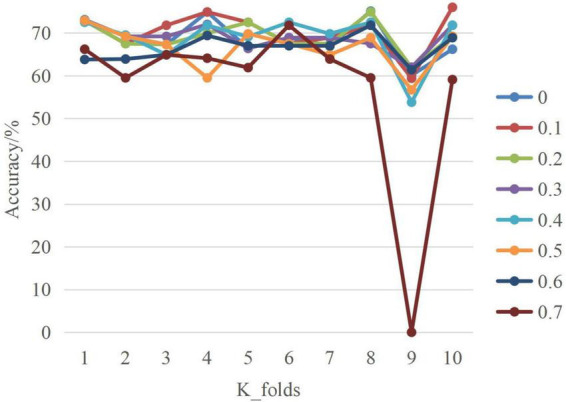
Line graph of SA-Bi-LSTM.

Figure 4 is the line graph of SA-Bi-LSTM network model. Where, the *x*-axis is the number of cross validation, and the *y*-axis is the accuracy of test set. This line chart shows the model accuracy varies with the number of cross-validation and dropout values. When dropout = 0.1, the model achieves the highest performance at different cross-validation. In addition, it can be seen that at the ninth cross-validation, the model presents the lowest inflection point and achieves the lowest performance under different dropout values, which may be related to data set division.

The performance of the SA-Bi-LSTM model on the dance database under different dropout values is shown in Table 4. It can be seen that when the dropout is not used, the model needs too many parameters to be trained, and the over-fitting phenomenon is serious. In other words, the model has achieved good performance on the training set and poor performance on the test set. When dropout is used and the value is large, too much dance emotion information is lost, resulting in a lower recognition rate.

**TABLE 4:** Accuracy comparison with different models.

Model	Accuracy/%
Support vector machine (SVM) (Sun et al., 2022)	57.61
SVM tree (Atila and Şengür, 2021)	72.92
CNN-LSTM (Shoeibi et al., 2021)	89.05
ACRNN (Tao et al., 2020)	94.78
A-BLSTM (Fujioka et al., 2020)	96.53
MHA (Nediyanchath et al., 2020)	96.58
SA-Bi-LSTM	97.96

Overall, when dropout = 0.1, the model achieves optimal performance on the test set, with an accuracy of 92.45% and precision of 94.11%, indicating that when dropout = 0.1, it not only prevents the over-fitting phenomenon, but most features are not lost.

Finally, Table 5 presents the comparison results of different methods in the field of emotion recognition in recent years. The accuracy of these studies is not well, which is mainly determined by the internal structure characteristics of dance data. In addition, the quality of data annotation further reduces the recognition accuracy of the data set. According to the annotation rules, only when the judgment of the emotion category contained in the audio clip is unanimously evaluated by more than half of the experts, can the clip be labeled. About 25% of the audio clips in the data set can not be assigned to the emotion label, and less than 50% of the labeled clips can obtain unanimous evaluation from all experts, which further illustrates the complexity of human emotion expression and the subjectivity of emotion evaluation. As can be seen from Table 5, compared with other comparison methods, the recognition performance of the proposed model in this paper is optimal on the dance data set. The recognition performance of traditional machine learning SVM is weaker than that of neural network. Sun et al. (2022), a single SVM with low-level feature set as input can obtain 57.61% accuracy. Fujioka et al. (2020), the ability of extracting potential relationships from original audio is far inferior to MHA-based models, and the reduction of computing efficiency brought by the increase of network depth is not conducive to its deployment in mobile terminals. Although the network structure of the model based on the manually extracted sound features such as Mayer’s spectrogram is simpler than the previous model, it is limited by the deviation of the artificial selection of sound features and the problems of high dimension and small scale of sample data. The model cannot mine the internal potential features of the sound sequence according to the characteristics of the specific dance emotion data set, and the generalization ability of the model is weaker than that of the MHA-based model. The experimental results show that the MHA-based model can effectively capture the internal temporal and spatial relationships in the original sound sequence. SVM as a classifier has a positive role in promoting the sound feature classification of high-dimensional small-scale samples.

**TABLE 5:** Confusion matrix with SA-Bi-LSTM.

	W	L	E	A	F	T	N
W	**97.75**	0	0	0.90	1.68	0	0
L	0	**95.17**	1.34	0	0	0	3.81
E	0	0	**93.59**	2.28	0	2.28	2.29
A	1.56	0	3.01	**95.76**	0	0	0
F	4.34	1.52	1.52	1.53	**90.25**	0	1.52
T	0	1.72	0	0	0	**96.88**	1.72
N	0	5.17	0	2.64	0	1.38	**91.25**

Bold value denotes the best value with proposed method.

## Conclusion

In this paper, a SA-Bi-LSTM model for dance emotion recognition is proposed, which has eight hidden layers, including two fully connected layers, convolution layer, jump layer, masking layer, Bi-LSTM layer, attention layer, and pooling layer. The convolutional layer in the model can effectively extract low-dimensional features of dance signals, and the attention mechanism can reduce the length of sequence information, fully mine the spatio-temporal structure information of dance signals, and further improve the recognition accuracy of dance emotion classification by combining SVM. The experimental results show that this model has greater advantages than the model based on Mayer spectrogram, because the input is original waveform and there is no manual feature extraction step, which brings convenience to the deployment of the model on the mobile terminal. In the future, we will continue to optimize the model, improve the accuracy of model recognition, and make the model have a development and application prospect in the mobile terminal.

## Data availability statement

The original contributions presented in this study are included in the article/supplementary material, further inquiries can be directed to the corresponding author.

## Author contributions

All authors listed have made a substantial, direct, and intellectual contribution to the work, and approved it for publication.

## Footnotes

1
http://www.bbci.de/competition/ii/#datasets
